# Spontaneous categorization of tools based on observation in children and chimpanzees

**DOI:** 10.1038/s41598-019-54345-1

**Published:** 2019-12-03

**Authors:** Thibaud Gruber, Aurélien Frick, Satoshi Hirata, Ikuma Adachi, Dora Biro

**Affiliations:** 1 Department of Zoology, University of Oxford, Oxford, UK; 2 Swiss Center for Affective Sciences, University of Geneva, Geneva, Switzerland; 3 Department of Psychology, University of Edinburgh, Edinburgh, UK; 4 Laboratory of Experimental Psychology, Suor Orsola Benincasa University, Naples, Italy; 5 Wildlife Research Center, Kyoto University, Kyoto, Japan; 6 Primate Research Institute, Kyoto University, Inuyama, Japan

**Keywords:** Human behaviour, Animal behaviour

## Abstract

The acquisition of the concept of ‘tool’ remains intriguing from both developmental and comparative perspectives. Our current model of tool use development in children is based on humans’ supposedly unique ability to adopt a teleological stance: the understanding of a demonstrator’s goal-based intentions when using a tool. It is however unclear how children and chimpanzees, our closest relatives, combine their knowledge of different objects whose function is to act on other parts of the environment, and assign them to a single category of ‘tools’. Here, we used a function-based approach to address this question. We exposed 7 to 11-year-old children and adult chimpanzees to a Matching-to-Function (MTF) task to explore whether they would sort tools and non-tools separately after demonstration of their function by an experimenter. MTF is a variant of Matching-to-Sample where the sample and the target are from the same category/kind rather than identical. Around 40% of children paired objects according to their function in the MTF task, with only one child younger than 8 years doing so. Moreover, when verbally questioned, these children offered a function-based answer to explain their choices. One of six chimpanzees also successfully paired objects according to function. Children and at least one chimpanzee can thus spontaneously sort tools into functional categories based on observing a demonstrator. The success of a single chimpanzee in our task suggests that teleological reasoning might already have been present in our last common ancestor but also shows that human children more readily conceptualize tools in a spontaneous fashion.

## Introduction

In our daily lives, tools are special artifacts whose nature as detached objects allows the user to modify other parts of the environment^1^. While long believed to be a hallmark of human uniqueness^2^, the discovery of tool use and making in wild chimpanzees (*Pan troglodytes*)^3^ fostered a growing interest in animal tool use behavior. To date, researchers have identified tool use in four phyla and 10 classes^1^.

Chimpanzees, as the most prolific non-human tool-users, represent a valuable model to study cognition underlying tool use^4^. Research has extensively explored their tool-using abilities, with a particular focus on behavioral variation suggestive of the existence of culture^5^. Evidence from both the wild and captivity has supported this claim by showing that chimpanzees are efficient social learners who acquire tool use information through observation^6,7^. Research has also investigated the extent to which individuals possess an understanding of the physical demands of and cause-effect relationships involved in tool use. For instance, field experiments in wild chimpanzees have shown that they are selective of both stone and leaf tools^8,9^, choosing those objects as tools that provide greater efficiency. However, wild chimpanzees also experience difficulties in understanding the properties of unknown tools^10^. Such results may generalize to other species as they are also found in, for example, wild capuchins (*Sapajus ssp*)^11,12^, raising questions regarding the general understanding of tools by non-human primates.

While research in the wild can bring much insight, theoretical considerations are necessarily limited by the constraints of the field, as many factors cannot be controlled^13^. Despite criticisms about their socio-ecological validity^14^, laboratory experiments appear more conducive to addressing certain aspects of chimpanzee cognition (see a review for primates in^4^). For example, tool-based tasks are in general more complex than non-tool-based tasks, which may have influenced previous interpretations of primate behavior^15^. While recent work has tackled the social side of tool-use transmission in primates in connection with the cultural claims, primate cognition related to the tools themselves remains poorly understood. For example, despite the fact that they are known to spontaneously sort aspects of their environment into categories^16^ it is yet unknown whether chimpanzees, and generally, other animals, are able to categorize tools as a special class of objects, that is, to distinguish them as objects that act on the rest of the environment, as humans routinely do. Determining how children and chimpanzees compare in their ways of conceptualizing tools appears crucial for two reasons: (1) reorganizing one’s knowledge, particularly of tools, is critical during development in humans^17^; and (2) such categorization is a building block of the phenomenon of culture as we envision it in humans, allowing one to modify the cultural repertoire of the species^18^.

Previous research with a bearing on such questions is scarce. In the late 1970s, Savage-Rumbaugh and colleagues introduced symbols for different types of ‘foods’ and ‘tools’ in their studies with captive chimpanzees^19^. Chimpanzees were able to sort objects according to their nature (‘food’ or ‘tool’) and use the symbols to request particular tools to recover particular food items from one another. However, these and subsequent studies (e.g.^20,21^) have been criticized as subjects’ performance could have resulted from training, rather than from spontaneous categorization^22^. In addition, the studies did not distinguish between objects that were tools and those that were not per se, with most objects used by chimpanzees being themselves tools. In other primate species, Santos *et al*.^23^ showed that cotton-top tamarins (*Saguinus oedipus*) and rhesus macaques (*Macaca mulatta*), two species that do not use tools in the wild, used perceptual features (e.g. color) to separate objects into categories such as tools or foods. Nevertheless, none of these studies addressed the question whether non-human primates are able to categorize tools *as* tools, that is, as meaningful ontological entities^24^ whose function is to act on the environment.

In comparison, the cognition surrounding artifacts – objects created or modified for a purpose, representing major components of human material culture – is well described in children^25,26^. Two-year-olds understand some properties of artifacts but do not form an overall concept of tools^27^, while three-year-olds understand that tools are ‘made for’ a given purpose and select them accordingly^28^. However, children only develop a ‘design stance’, that is they understand that tools are intentionally manufactured by a designer to fulfill some function, around six years of age^29^. Kelemen and Carey^29^ suggest that children may start acquiring a causally rich explanatory structure represented by an intentional-historical design stance based on intended function as early as two years of age, as long as the perceptual information is consistent with a specific function, leading them to categorize artifacts on the basis of functional properties. This represents an important cognitive and representational shift, from age five where the function of an artifact is not completely clear in the child’s mind, fulfilling any goal a user might have, to age seven where the function has become that of the artifact’s typical or intended use^30^. A major theory for tool use acquisition that has gained traction over the past decade is the teleological stance^29,31,32^. There is evidence that from two years of age, children build on their developing intentional and mind-reading abilities to attribute a given function to an artifact^28,29^. In other words, tools are ‘for’ something, and this can arise after a single exposure to an adult apparently intentionally using a novel tool^33^. Nevertheless, little is known about how such knowledge forms in the developing child, and particularly when ‘tools’ come to be recognized as part of a specific class of objects that can act on other parts of the environment. In comparison, chimpanzees are argued to be unable to adopt a teleological stance^31^.

In the present study, we aimed to address the question of the representation of tools *as* tools in both children and chimpanzees. To do so, we relied, as described above, on the fact that tools are understood to be objects that perform a function in the course of which they modify their environment, and are used intentionally by a user to do so. In contrast, non-tools are those objects whose manipulation by a user does not result in a modification of the environment. We tested both adult chimpanzees and 7 to 11-year-old human children following the same protocol. Taking advantage of the advanced social learning abilities of the two species, we hypothesized that demonstrating the function of novel tools (and, conversely, the absence of function of non-tools) in front of participants may lead them to spontaneously categorize some objects as ‘tools’ and others as ‘non-tools’. After observing the experimenter engaging with these objects in a ‘Demonstration Phase’, participants engaged in a classic ‘Matching-To-Sample’ (MTS) paradigm where they had to pair one of two possible alternatives with a given sample. Crucially, while most of the trials were based on identity (with the rule being to find among the alternatives the object identical to the sample), we also introduced probe trials called ‘Matching-To-Function’ (MTF), where the two alternatives were a tool and a non-tool, and the sample either a tool or a non-tool, but *not identical* to either of the alternatives. We predicted that if subjects spontaneously categorized objects according to their observed function, the probe trials should yield pairings between tool/tool and non-tool/non-tool at a rate higher than random. In contrast, if tools and non-tools were paired more readily according to their perceptual features, this would indicate that subjects were following a perceptual rule for pairing objects. Finally, if the pairing pattern did not differ from random, this would indicate that the subjects failed to construct any rule, or failed to follow it. As such, only the first outcome would yield strong results with respect to spontaneous categorization abilities for tools.

## Materials and Methods

### Subjects

Six chimpanzees (*Pan troglodytes*) participated as subjects, one male and five females (Ai, female, age 41 years; Chloe, female, age 37; Pendesa, female, age 40; Ayumu, male, age: 17; Cleo, female, age 17; Pal, female, age 17), housed at the Primate Research Institute of Kyoto University in Inuyama, Japan. The chimpanzees lived in a group of 13 individuals with access to indoor compounds and a 770 m^2^ environmentally-enriched outdoor enclosure equipped with climbing frames, small streams and various tree species^34,35^. They were fed twice daily with a wide variety of fresh fruits and vegetables supplemented with nutritionally balanced biscuits. Water was available ad libitum. All participants had extensive previous experience with various cognitive tasks, including computerized Matching-to-Sample tasks^36,37^ and tasks where they handled physical objects^38^ and tools^39,40^. They all participated in the present experiments voluntarily and could stop at any time.

The human children participants were all recruited from the same primary school in Orléans, France, and belonged to classes between the 2^nd^ and the 5^th^ grade. Informed consent was obtained from a parent and/or legal guardian prior to participating in the experiment, following advertising by the head teacher. We tested a total of 71 children (mean age ± SD = 9.07 ± 1.24 years, age range = 7.17–11.5 years, 31 females, 40 males), whom we sorted into four age classes: 7–8, 8–9, 9–10, and 10–11 years, roughly equivalent but not identical to the grades they were in. We excluded six children prior to analysis, either because they did not complete the experiment in its entirety or because there were too few (older) children in their age class to run meaningful comparisons. Experiments with chimpanzees were run between March and November 2017. Experiments with children were run between March and April 2018. All protocols were reviewed and approved by the Primate Research Institute of Kyoto University and the Ethics Committee of the Faculty of Psychology and Educational Sciences at the University of Geneva. All methods were performed in accordance with the relevant guidelines and regulations, in particular the ASAB/ABS Guidelines for the Use of Animals.

### Materials

**Figure 1:**
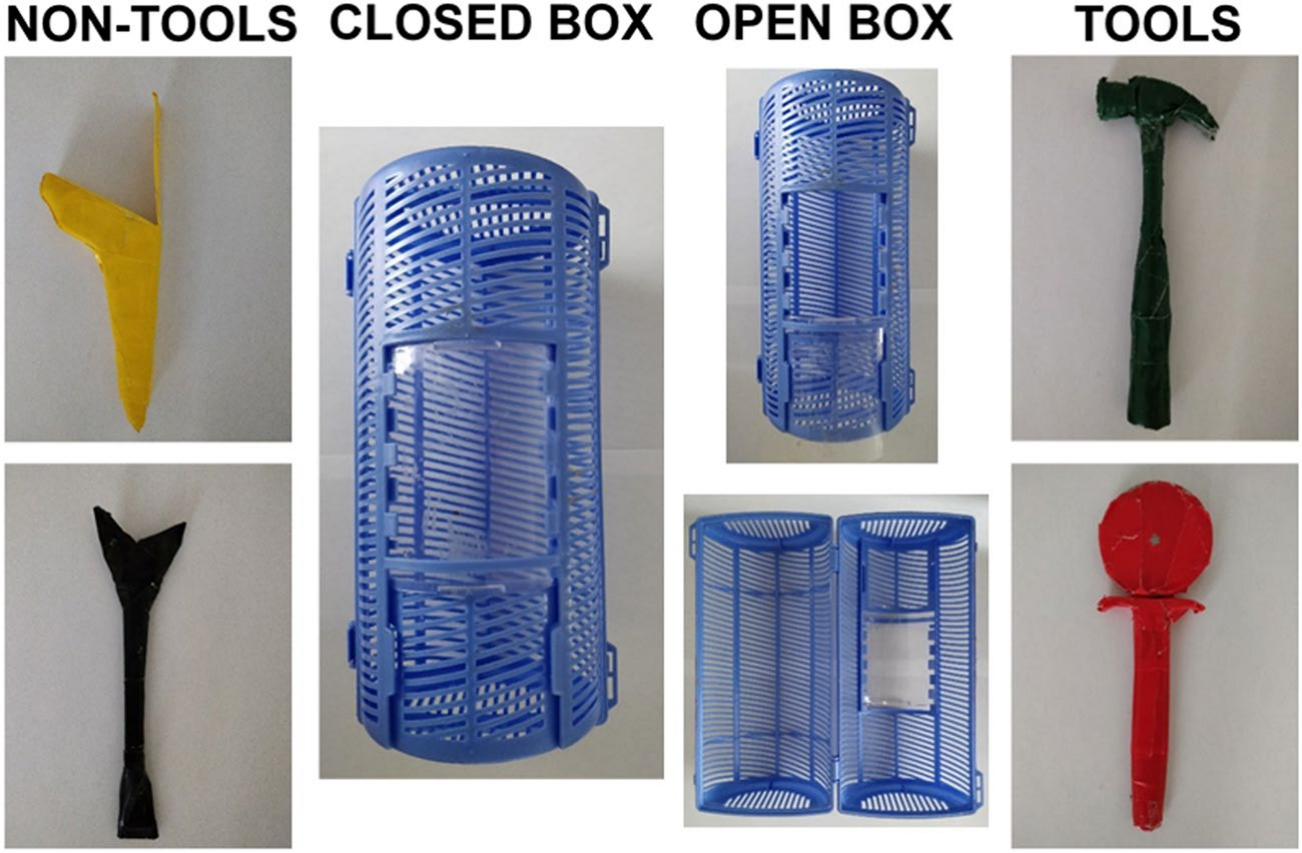
The box and objects used in the experiment with children and chimpanzees. Left: non-tools, the wedge (W) and the stick (S). Right: tools, the hammer (H) and the pizza slicer (P). Center: box closed (left) and opened through two mechanisms (top: window down; bottom: hinge open).

We modified a blue plastic cylinder box (23.5 × 10.5 cm) such that it could be opened in two different ways: either by sliding open a transparent window (8 × 6.5 cm), or by cutting through masking tape holding its two halves together. We used four objects with different affordances, all covered in insulation tape of different colors (Fig. 1). Two objects were used as tools (that is, they were used to open the box): a hammer (H, 19 × 2.5 × 6.5 cm, green tape) used to push down the transparent window, and a pizza slicer (P, 19 × 2 × 6.5 cm, red tape) used to slice open the masking tape and open up the box along its hinges. Two further objects were used as non-tools (that is, they were only put in contact with the box but did not cause it to open): a Disney spoon transformed to look like a stick (S, 19 × 2.5 × 5 cm, black tape), and a door wedge extended with cardboard to match the length of the other objects (W, 19 × 2 × 7.5 cm, yellow tape). Each object was of a different color to limit perceptual biases in pairing. For chimpanzees, we placed frozen berries, a desirable treat, into the box. For children, we placed tokens inside the box that could be traded at the end of the experiment for stickers.

### Protocol

**Figure 2:**
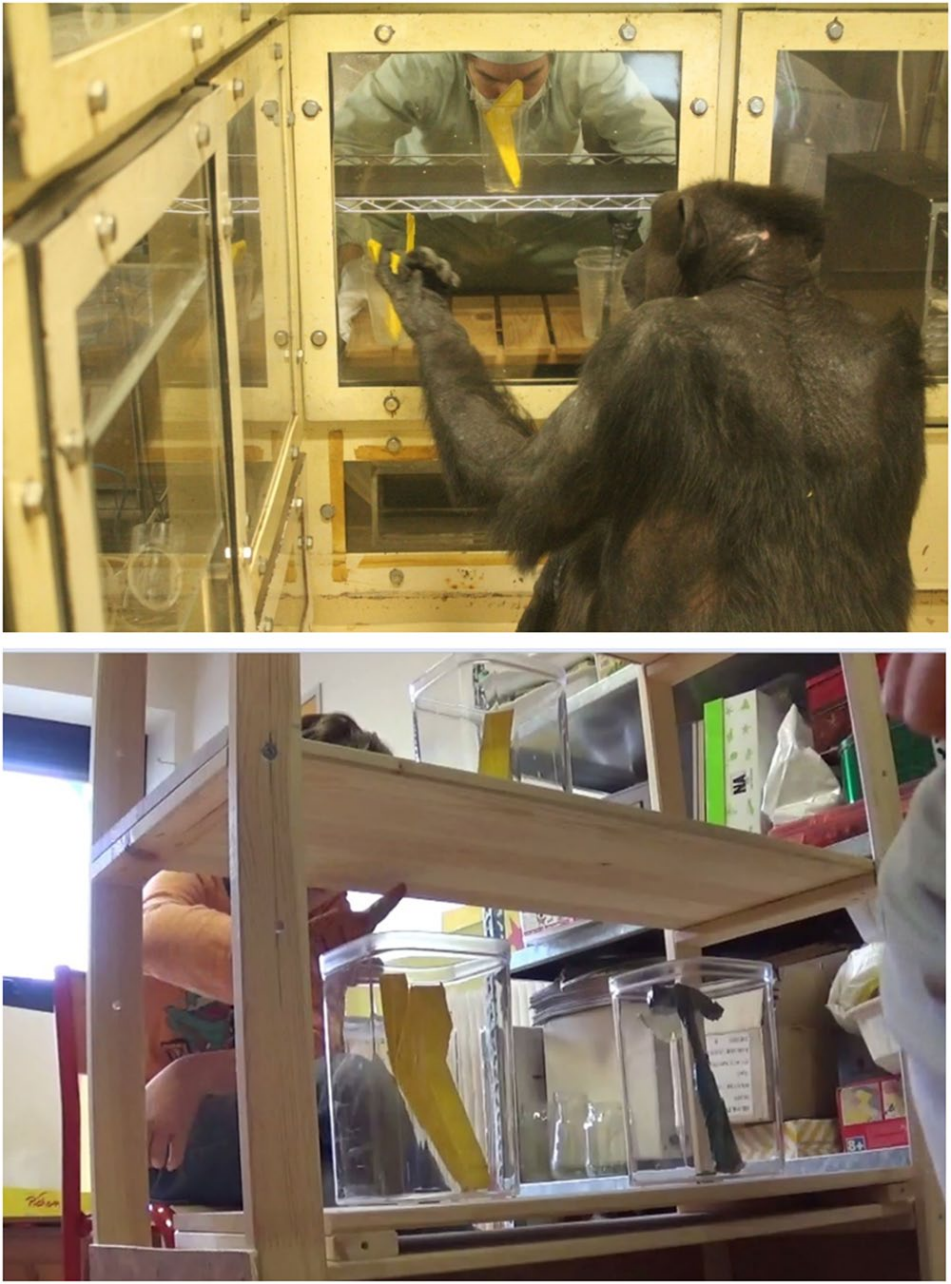
Chimpanzee subject (Ai) and a child participant in the identity Matching-to-Sample phase of the experiment. Samples are presented in compartment 3 of the apparatus; the two alternatives (one of which is identical to the sample) are located in compartment 2. Subjects can be seen making their choice between the alternatives presented, through pointing.

Both children and chimpanzees were invited to sit opposite the experimenter who was seated behind the experimental apparatus (Fig. 2): a metal (for chimpanzees) or wooden (for children) shelf unit measuring 120 × 66 × 26 cm and consisting of four compartments (height of compartments from bottom to top: 1: 34.5, 2: 29, 3: 29 and 4: 24 cm). Experiments with chimpanzees were conducted inside dedicated testing booths with transparent acrylic walls^37^. Chimpanzee subjects were called in individually by name from their large outdoor enclosure, and proceeded to the testing booth voluntarily. For chimpanzees, the shelf was positioned next to an acrylic window on the wall of the booth. In addition, below the window and installed in the lowest compartment (compartment 1), an acrylic tube allowed the provision of food rewards. For children, the shelf unit was positioned in the center of a room adjacent to their classroom, where children were invited to join the experimenter. An experimental session comprised two phases: a demonstration phase, and a MTS phase. In the demonstration phase, subjects observed the experimenter handling the objects and the box, while in the MTS phase, they were required to make choices between two objects presented in compartment 2 based on a sample object provided in compartment 3. All remaining objects were held in the top compartment (4), out of sight of both chimpanzees and children. The order of presentation of objects was counterbalanced across demonstration phases, as well as during the MTS task such that the correct answer was on the right in half of the trials, and on the left in the other half. All possible combinations of sample/alternatives were present and counterbalanced across the 36 trials of a given session. Each testing session lasted approximately 20 minutes, with a maximum of two sessions conducted per chimpanzee per day. All participating children were exposed to two consecutive sessions, for the same duration as the chimpanzees (see below for protocol differences).

#### Demonstration phase

At the start of each session, the subjects observed the experimenter (TG for chimpanzees, AF for children) using four objects in relation to the blue transparent box. The demonstration took place in compartment 3 of the apparatus. Two of the four objects were used as tools (H to open the transparent window on the box, and P to slice open the masking tape holding the box together; see Supplementary video S1 for demonstrations). Two additional non-tool objects (S and W) were simply made to touch the box in two different places and were held against it for the same amount of time as the tools while the box remained closed (see video S1). After each demonstration, participants received a reward (a frozen berry for a chimpanzee, and a token for a child) and the object used by the experimenter was removed from the shelf. The reward was either recovered from inside the box (in the case of demonstrations involving tools, whose use always resulted in the opening of the box) or taken from a container next to the experimenter (in the case of non-tools, whose use never resulted in the opening of the box). The consumption of the berry reward by chimpanzees, or the obtaining of a sticker by children, signified the end of the demonstration for that object for both tools and non-tools, and the start of the demonstration of the next object, or the start of the MTS phase. Following the demonstration of all objects once each, the participant entered the MTS phase of the session.

#### MTS phase

Immediately after the observation phase, subjects were given a simple MTS task (Fig. 2). The experimenter presented a sample (one of the four objects that subjects saw him engaging with in the observation phase) in a transparent plastic jar in the upper compartment. The experimenter subsequently displayed a forced choice between two additional transparent jars containing another copy of the sample (the correct answer) and a distractor (one of the three remaining objects) on a tray placed on compartment 2. Subjects were required to point to one of the alternatives. For chimpanzees, correct answers were rewarded with a piece of food and the playback of a particular sound (‘Excellent’), while incorrect answers were not rewarded and chimpanzees heard a different sound (‘Doh!’). For children, correct answers were rewarded with an affirmative response from the experimenter (‘Oui!’/*yes*), and incorrect answers were commented upon with a negative response (‘Non!’/*no*). Whenever an incorrect choice was made, the trial was repeated (correction trial). At the first incorrect answer, the experimenter removed the tray containing the two choices, tapped on the jar holding the sample, and presented both alternatives again. If the subject again gave a wrong answer, the experimenter removed the tray, took out the distractor, and presented the tray again but with only one object (the correct one). This protocol allowed us to train chimpanzee and human subjects on identity MTS with no additional verbal cues from the experimenter. This protocol was maintained through a series of 12 consecutive baseline trials. After 12 consecutive trials, the subject was exposed to two novel series of 12 additional trials (24 in total), where there were no more correction trials. In addition, we included eight Matching-to-Function (MTF) probe trials among the last 24 MTS trials, distributed pseudo-randomly through the sequence (i.e. a total of 16 baseline identity MTS trials and 8 MTF probe trials, constructed so that the alternatives always contained one tool and one non-tool, covering all possible pairings and spatial configurations). Probe trials differed from baseline trials in that the correct answer according to functional matching was to pair a tool sample with the second tool, while a non-tool sample was to be paired with the second non-tool. Because we wanted to avoid training associations between given pairings, none of the probe trials was rewarded. Both chimpanzees and children heard a different sound (a neutral tone for chimpanzees, and ‘Je vois’/*I see* for children).

### Difference in protocol between chimpanzees and children

Each child was exposed to two consecutive sessions of the experiment, each including the Demonstration phase and the MTS phase. For the Demonstration phase, the order of demonstrations was identical between the two sessions for any given subject, but differed between subjects. For the MTS phase the probe trials were counter-balanced so that, by the end of the 16 trials, each combination of tools and non-tools, once each on the right and on the left, would have been observed by a child participant. Finally, at the end of the two sessions, we asked children what rules they had followed when making their choices in the MTS phase (‘Quelles règles as-tu suivies durant ce jeu?’/*What rules did you follow during this game*?). This way, we could relate their performance in the probe trials with verbal reporting of the rule they constructed for the task, for both the MTS identity and the MTF probe trials.

In comparison, because of the small number of participating chimpanzees, each subject was exposed to 16 sessions of the experiment, with a maximum of two consecutive sessions on a given experimental day, in order to complete four repetitions of the different stimuli pairings. Consecutive demonstrations presented the four objects in different orders (for example, H, P, W, S in Session 1, W, S, P, H in Session 2, etc) to prevent chimpanzees from learning a particular sequence and failing to pay attention to all demonstrations. Finally, prior to the 16 test rounds, we trained the chimpanzees to perform MTS with real-life objects (different from those used in the experiment proper). This was because although each subject was highly experienced at MTS using stimuli presented on computer touchscreens, they had not matched 3D objects previously. First, chimpanzees were familiarized with real-life-object identity MTS over 16 experimental days with sets of balls of different colors. Because none of the chimpanzees acquired the MTS rule, we subsequently used a range of objects of various shapes and colors. For both training phases, chimpanzees received three series of 12 trials consisting of identity-based baseline trials (the first series including correction trials, but not the two subsequent series). Only when chimpanzees successfully completed either three consecutive baseline sessions with over 80% correct answers, or two consecutive baseline sessions with over 85% correct answers on the last two series (first criterion) were they allowed to proceed to a MTS task including the objects they later saw demonstrations of, where they had to once again either complete three consecutive baseline sessions with over 80% correct answers, or two consecutive baseline sessions with over 85% correct answers on the last two series (second criterion). Once this step was reached, they could proceed to experimental sessions including MTF probe trials. Children had no such MTS training as almost all participants acquired the MTS rule within a few trials (first to third trial of the first session) apart from two subjects whom we excluded from data analysis (see below).

### Data analysis

We recorded subjects’ first choices in each trial. Because of the small number of chimpanzee subjects passing the two criteria and the variation in their choice behavior, we analyzed their results at the individual level. For each subject, we performed a binomial test to evaluate whether, in probe trials, the number of function-based pairings of sample and alternative was significantly higher than expected by chance. For children, we first compared the proportion of children in each age class (7–8, 8–9, 9–10 and 10–11) who verbally indicated a functional rule for pairing, using a Chi-squared test. We also tested for a sex effect across age classes in the number of correct trials with a t-test after checking for normality. Finally, we investigated the propensity of children to verbalize a function-based rule, using a Generalized Linear Mixed Model (GLMM) with a logit link function and binomial error distribution with three fixed factors (Age, Sex, Number of function-based pairings) and Participant ID as a random factor. We also tested a model including the interaction between Age and Sex, but as we did not find an effect of this interaction, we removed it from the final analysis. Using SPSS 25.0, we first tested our model against a model including only the intercept using an F-test. This tested whether the model as a whole accounted for a significant proportion of the variance in the dependent variable (whether children verbalized a function-based rule, yes/no). We subsequently looked at the effect of each individual factor with likelihood ratio tests using t-tests. For both chimpanzee and human participants, we only included sessions with overall more than 80% of success in the baseline trials in the last two series. For this reason, we removed one test session for one of the chimpanzee subjects (Pendesa), and all the data from two children who did not perform above 80% success in the baseline trials across either of the two sessions they participated in (leading to a sample size of N = 63, with six participants excluded pre-emptively and two subsequently at analysis stage).

## Results

### Chimpanzees

Three chimpanzees (Ai, Chloe and Pendesa) passed both the first and second criteria (identity MTS with objects not used in the experiment proper, and with objects later used in the experiment proper, respectively). Ai passed the first criterion after seven sessions, and the second after three sessions. Chloe passed the first criterion after seven sessions, and the second after two sessions. Finally, Pendesa passed the first criterion after 15 sessions, and the second after two sessions. Two chimpanzees (Pal and Ayumu) did not participate in enough sessions to acquire the main rule of the MTS, and one chimpanzee (Cleo) failed to acquire it altogether within the timeframe of the experiment, that is, none of these three chimpanzees obtained more than 80% on several consecutive baseline sessions to reach the first criterion, and hence did not progress further in testing.

**Figure 3:**
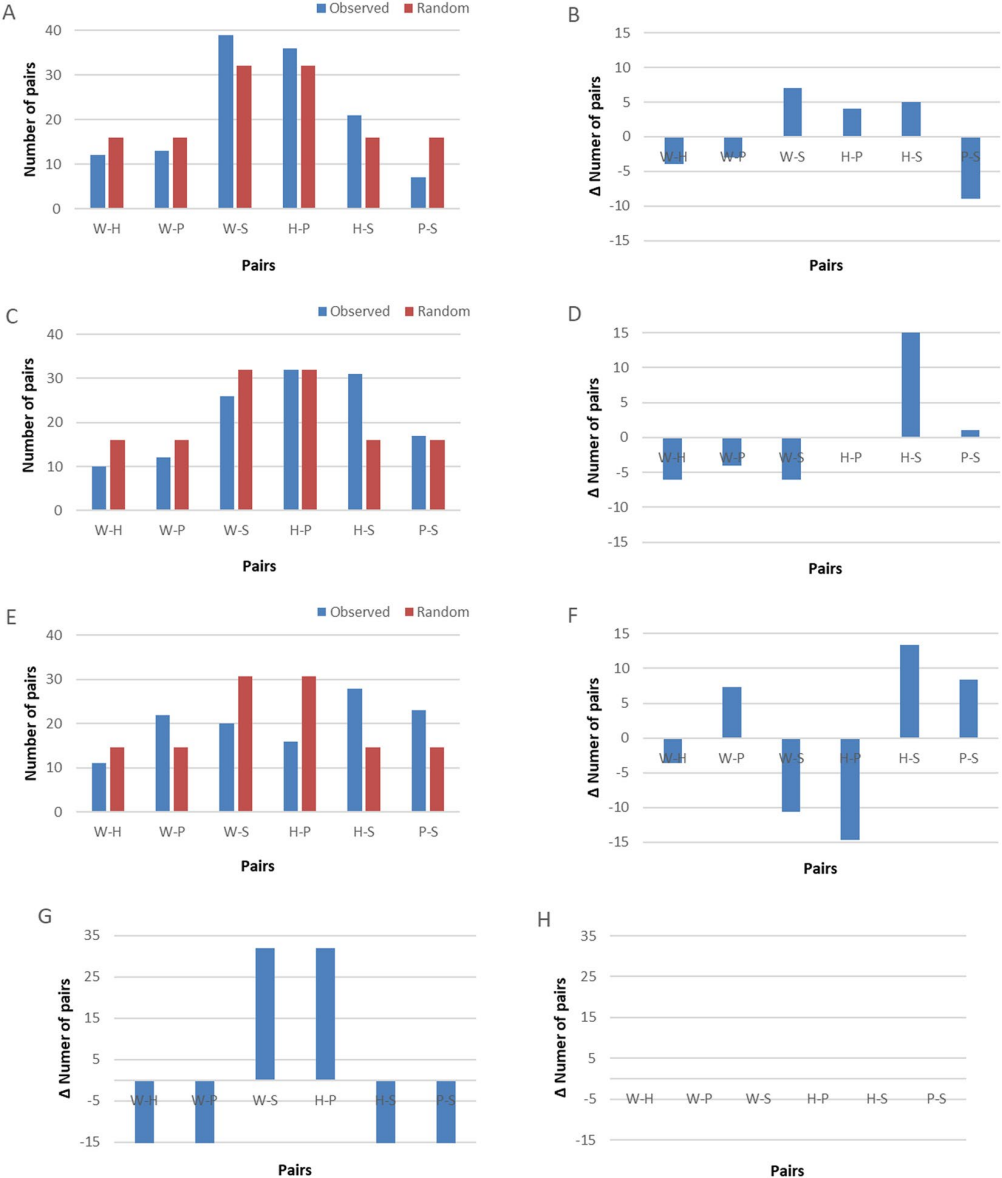
Pairing results in probe trials for the three chimpanzee subjects that progressed to the MTF phase (**A,B)**: Ai, (**C,D)**: Chloe, (**E,F)**: Pendesa). Panels B, D, F show the distribution of results obtained after subtracting the random distribution for 128 or 120 trials (depending on subject) from the observed results obtained by each chimpanzee. G and H show expected distributions after subtraction for fully function based (**G**) and fully random (**H**) pairing of the objects (128 trials, W = Wedge, H = Hammer, P = Pizza cutter, S = Stick) respectively. In particular, for panel H, because the forced-choice always involved at least the other tool or non-tool (i.e. W with S; and H with P), paired with one of two distractors, there are twice as many chances to hit the correct pairing by chance, hence double the distribution for the pairs W-S and H-P.

Ai and Chloe subsequently completed 16 sessions (128 probe trials), and Pendesa 15 sessions (120 probe trials), with over 80% correct answers in baseline trials. Binomial tests were used to calculate the probabilities of scoring as many or more correct trials (out of the total presented) as those scored by each subject: Ai paired objects according to function in 75 of 128 probe trials (58.6%, p = 0.032, probability of exactly or more than 75 cases, Fig. 3A,B). Chloe in 58 of 128 probe trials (45.3%, p = 0.87, probability of exactly or more than 58 cases, Fig. 3C,D), and Pendesa in 36 of 120 probe trials (30%, p < 0.0001, probability of exactly or *less* than 36 cases out of 120, Fig. 3E,F). The distribution of these three chimpanzees’ choices is compared in Fig. 3.

### Children

**Figure 4:**
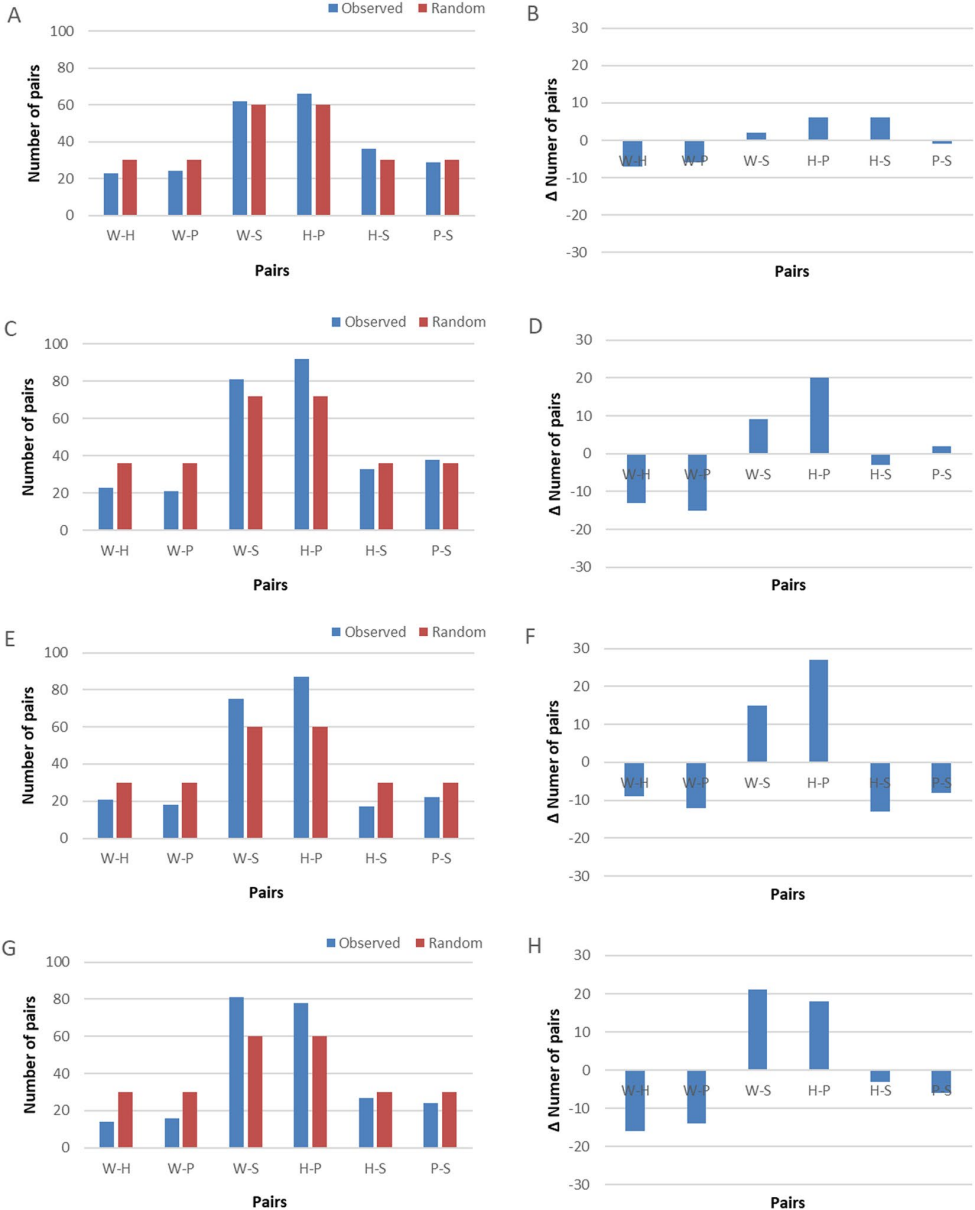
Pairing results in MTF probe trials in the four age classes of children tested (7–8 y: (**A**,**B**); 8–9 y: (**C**,**D**), 9–10 y: (**E**,**F**), 10–11 y: (**G**,**H**); W = Wedge, H = Hammer, P = Pizza cutter, S = Stick). This figure shows cumulative data for each age group, i.e., all trials across all subjects in the given group pooled together. Panels B, D, F and G show the distribution of results obtained after subtracting the random distribution for the respective number of trials corresponding to the number of tested children per age class (see legend of Fig. 3 for explanation).

The spontaneous pairing of objects according to function increased between 7 and 11 years of age. Only one child out of 16 (6%) in the 7–8 y age class correctly offered a rule based on tool function, compared to 8 out of 18 (44%) in the 8–9 y class and 8 out of 15 (53%) in both the 9–10 y and 10–11 y classes. The reporting of function-based explanations differed between age classes (Chi-squared test, χ^2^(1) = 9.34, p = 0.025, Table 1). 7 to 8-year-olds paired objects according to function in 128 of 240 probe trials (53.3%), 8 to 9 years-olds in 173 of 288 probe trials (60.1%), 9 to 10 years-olds in 162 of 240 probe trials (67.5%), and 10 to 11 years-olds in 159 of 240 probe trials (66.3%, Fig. 4, Table 1).

**Table 1:** Children’s performance in the MTS task.

Age Class	7–8 y	8–9 y	9–10 y	10–11 y	Total
Number of children (Female, male)	15 (5,10)	18 (11,7)	15 (7,8)	15 (4,11)	63 (27,36)
Number of children offering a function-based rule (proportion)	1 (0.07)	8 (0.44)	8 (0.53)	8 (0.53)	25 (0.38)
Proportion of males offering function-based rule	0	0.29	0.5	0.45	0.31
Proportion of females offering function-based rule	0.2	0.55	0.57	0.75	0.52
Mean number of function-based pairings (out of 16) by subjects offering function-based rule	13	12.88 [9–16]	13.25 [8–16]	12.75 [11–15]	12.96
Mean number of function-based pairings (out of 16) by subjects not offering function-based rule	8.2 [6–14]	7 [4–10]	8 [3–13]	8.14 [5–14]	7.84
Mean proportion of function-based pairings	0.53	0.60	0.68	0.66	0.62

The table reports the proportion of subjects in each age and sex class who gave a function-based rule for the task during verbal reporting at the end of experimental sessions, and their accuracy in correctly pairing according to function during MTF probe trials. Values in square brackets indicate range.

Children who offered a functional rule for pairing displayed on average 13 (out of a total of 16 presented, range: 8 to 16) function-based pairings during MTF trials, compared to an average of only 8 (range: 3 to 14) among children who did not offer this rule (Table 1). There was nonetheless large variation across age classes, with some children offering the function-based rule and yet only displaying 8 (N = 1) or 9 (N = 2) function-based pairings, while others did not offer a function-based rule despite displaying 13 (N = 1) or 14 (N = 2) correct function-based pairings. We also found a sex difference in that girls started offering a function-based explanation at an earlier age than boys (t test, t = 2.24, df = 23, p = 0.035; Table 1). In comparison, chimpanzee Ai made 10 out of 16 functional pairings in her first two sessions, Chloe eight, and Pendesa four.

We ran a Generalized Linear Mixed Model with Age (in months, continuous), Sex (male, female) and number of function-based pairings as fixed factors and participant ID as a random factor to characterize the spontaneous generation of a function-based rule by children. This model was significantly different from the model including the intercept only (F = 6.50, df1 = 3, df2 = 59, p = 0.001, Table 2), showing that our model accounted for some of the variance in our dependent variable (offering a function-based rule). In particular, we found that the number of function-based pairings significantly influenced generating a function-based rule (t = −4.039, p < 0.001). In comparison, Age (t = −1.403, p = 0.17) and Sex (t = 0.784, p = 0.44) did not. That is, the higher the correct number of function-based pairings during MTF probe trials, the more likely the child would subsequently offer a function-based pairing rule for his or her choices.

**Table 2:** Results of the GLMM analysis for generating a function-based rule in children.

Model Term	F	p	Coefficient	Standard Error	t	95% Confidence Interval	
						Lower	Upper
Intercept	6.50	0.001	10.98	3.55	3.092	3.87	18.078
Age (months)	1.97	0.17	−0.041	0.029	−1.40	−0.099	0.017
Sex	0.62	0.44	0.62	0.79	0.78	−0.96	2.20
Function pairs	16.31	0.000	−0.62	0.16	−4.04	−0.93	−0.32

Interestingly, children who did not offer a function-based rule also varied in terms of the rules they declared they were following. Most of the children simply refrained from discussing the rule in the probe trials, while accurately explaining the identity MTS rule. This was especially apparent in the youngest age classes, where over half of the children who failed to give a function-based rule altogether failed to address the probe trials in their discussion of the rules; however, failing to mention the probe trials was also prevalent among the oldest children (Table 3). Some children stated that they did not understand the probe trials while others replied that they chose at random or that they followed complex rules (‘Other’) in their choices (for example, one child offered the justification that she was aiming to pick the object whose function had been demonstrated after the sample during the demonstration phase). Finally, perceptual-based rules appeared mostly in older children, but remained rare (Table 3).

**Table 3:** Distribution of verbal responses given by children who did not offer the function-based rule.

Age class	N	No function-based rule	Fail to mention	%	Did not understand	%	Chose at random	%	Perceptual-based rule	%	Other	%
7–8	15	14	7	0.50	3	0.21	2	0.14	0	0.00	2	0.14
8–9	18	10	6	0.60	0	0.00	2	0.20	1	0.10	1	0.10
9–10	15	7	2	0.29	1	0.14	1	0.14	2	0.29	1	0.14
10–11	15	7	3	0.43	0	0.00	2	0.29	2	0.29	0	0.00

## Discussion

While tool use cognition has been at the center of the debate regarding the uniqueness of the human mind^5,41^, to date there is little discussion of the conceptual underpinnings of tool use from a comparative perspective^24,32^. Hence, we still do not know whether tool-using nonhumans represent tools the same way we do, that is, minimally, as objects that function on other parts of the environment. One promising avenue of research in developmental psychology has been to focus on the functional characteristic of tools compared to other objects, and the way a demonstrator conveys their usefulness to attain a goal^31,42^. In the present study, we compared captive chimpanzees and human children in a MTS task, investigating whether they would spontaneously sort together objects that they had seen being used by a demonstrator as tools to open a box against objects that did not have such a function. We found that one chimpanzee (Ai) and around 40% of all tested children indeed spontaneously paired these objects together significantly more often than other non-functionally matched pairs. We discuss our results from both a comparative and a developmental perspective.

Our results, both in chimpanzees and human children, show that our task was challenging, with less than half of the children overall and only one chimpanzee tested successful. Several explanations could be proposed for this. First, there was no reinforcement for ‘correct’ pairing in probe trials, meaning that subjects received no feedback regarding their choices. Indeed, there was no correct answer, as we were interested in the spontaneous sorting behavior of the two species. However, despite the lack of reinforcement, some subjects in both species preferentially sorted objects based on their function. Given that for children we were able to verify that their sorting behavior was indeed based on a function-based rule through a post-test verbal interview, it should be possible to extend this reasoning to the one chimpanzee (Ai) who also paired according to function. While we note that Ai’s performance was only slightly (though significantly) above chance, we also note that the GLMM run with the children’s data found that the only predictor in our model of successfully offering a function-based rule at the end of testing was the number of function-based pairings a subject had produced earlier. As such, since we relied on the same predictor to analyze the chimpanzees’ and children’s results in the same task, and that only the number of function-based pairings emerged as a significant factor in the children’s case, an above-chance number of function-based pairings by Ai suggests that her performance may also have been supported by a function-based rule. The only difference in protocol was that chimpanzees were tested over multiple sessions, which could have indirectly acted against function-pairing and favored random-pairing. Specifically, the repetition of sessions might have allowed chimpanzees to learn that the probe trials were never rewarded and therefore might have caused them to become unmotivated and thus to choose at random. Whether or not chimpanzees lacked motivation once they had seen that probe trials were never rewarded, our protocol prevented associative learning in probe trials and did not give an incentive to subjects of either species to improve between trials. Examining the results of chimpanzee subjects in the first two sessions only, we found that Ai (10 correct trials out of 16), Chloe (8) and Pendesa (4) broadly matched their later results. In particular, Ai’s results in the first two sessions, while lower than the average for children in the 8–9 y and 9–10 y age classes, were above some of the children in these two age classes who offered a function-based rule.

Overall, our results also suggest that chimpanzees might have been more influenced by perceptual biases than children. One particular pairing, the dark green hammer (tool) with the black stick (non-tool), might indeed have been strengthened by perceptual biases, especially for chimpanzees, as the two objects could have appeared quite similar through the acrylic glass (TG, personal observation, Figs. 1 and 2). This pairing was found at a higher rate than expected in the results of all chimpanzees (Ai, Chloe and Pendesa), but also in children in the 7–8 y, 8–9 y, and 10–11 y age classes, showing that they exhibited some of the same possible biases as chimpanzees. Nevertheless, it remains unclear whether this is truly a perceptual bias. First, there were surprisingly few children who proposed a perceptual-based rule as the rule they followed to pair objects, showing that they either were not aware that they did follow such a rule, or that this rule was not important to them. Such a result is interesting in light of the ontogeny of category formation in children, suggesting that the formation of the concept of ‘tool’ may follow a different pathway to that of non-tool objects or animate beings, which first follow a perceptual-based conceptual build-up^27^. In addition, one chimpanzee subject (Pendesa) frequently displayed the hammer-stick pairing, but she also frequently paired the red pizza cutter with the black stick (P-S), where there was no evident perceptual commonality between the two objects. Because Pendesa took twice as long as the other chimpanzees to acquire the MTS rule and went through several phases of biases during learning (e.g. side biases), we cannot exclude that she also relied on particular idiosyncratic rules in her subsequent pairing behavior. While more chimpanzees would have been needed to quantify chimpanzee pairing behavior in more detail, the presence of three different pairing patterns (function-based, random, and possibly percept-based) in our three subjects suggests that chimpanzees are able to sort objects based on different rules. In particular, Ai’s results, being largely function-based, are interesting for discussions of goal-oriented actions. In summary, our results with chimpanzees are weaker than for children, resulting in only 17–33% (1 of 6 to 1 of 3, depending on the pool considered) of our chimpanzee participants sorting tools with tools and non-tools with non-tools, compared to 40% of all children. Nevertheless, our experiment also suggests that at least one chimpanzee was able to sort together objects used to act on other parts of the environment, a weak but significant result suggesting that she perceived them as different from other objects.

Our results are also interesting from a developmental point of view. It is indeed surprising that less than half of our tested children succeeded in the task overall, despite all having developed a conceptual understanding of tools by the time they participated in our experiment according to existing literature^29,30^. Indeed, the tested children should have, by age seven, displayed evidence of the full-fledged understanding of tools as particular objects of the environment. This appeared to be the case, as even some of the youngest participants referred to *all* the experimental objects as ‘tools’ (‘outils’) when discussing them and most participants referred to the hammer or the pizza cutter as objects they were familiar with in their normal day-to-day life. Despite this fact, which could have arguably assisted them in sorting these objects together based on familiarity (a rule that was never given by any tested child), many participants failed to sort by function. We discuss below several possible reasons.

A first possibility is that there was no clear difference between the tools and the non-tools in our experimental setting. For example, both human and chimpanzee participants might have considered that the ‘non-tool’ demonstrations were in fact instances of unsuccessful tool-use (as the non-tool objects were also put in contact with the box), and this could have impacted their sorting behavior. We would argue against this interpretation. Indeed, our paradigm relied on the assumption that a tool is defined by its function (in this case ‘opening the box’). All demonstrations were similar in duration (see Supplementary video S1), and they all were rewarded, independently of whether the box was opened or not, so that all demonstrations of object manipulation could be assumed to be completed upon receiving this reward. On a more theoretical level, we may argue that holding such a distinction between tools and tools-that-are-not-successful in itself constitutes a complex mental construction, which may go beyond the simpler representation of some objects allowing the opening of a box and others not. In effect, if one considers that the task involves a hierarchy of goals: (1) ‘retrieve the reward inside the box’, (2) ‘open the box’, and (3) ‘open the window’/‘slice the tape’, our ‘non-tools’ already fail both the first and second order goals, while our ‘tools’ only get distinguished at the level of the third order. This also suggests that the particular design of our experiment may have required our participants to distinguish objects only up to the second level (open the box: yes/no, that is the functional level or end result) – a level that does not need to distinguish according to the method used. Interestingly, this distinction between action and result learning is also found in the social learning literature where chimpanzees are known to engage in end-result copying emulation learning but where action copying imitation appears limited and is more controversial^6,43^.

Another possibility lies in the phenomenon described as *functional fixedness*: that is, already being familiar with the function of some of the objects could have prevented subjects from closely observing their function in the experimental context, and hindering their sorting behavior^44^. However, functional fixedness is positively correlated with age^43^ and the pattern that would be expected was not apparent in our data, as older children were the ones most likely to utilize the demonstrated functional aspect as a clue for sorting objects. A third possibility is that despite having already much of the conceptual cognitive machinery present^27,29^, children are yet to truly understand the concept of tool and what it entails (inclusive of some objects, but not of others). They may also need more exposures to the use of a given object to extrapolate its function rather than a limited number of demonstrations. Interestingly, girls in our experiment appeared to pick up the function-based rule at a younger age than boys. This is in line with both experiments in children where girls tend to copy meaningful actions more faithfully^45,46^ and natural observations in chimpanzees and bonobos where females appear to pay attention to conspecifics’ tool use more closely than males^47,48^. Altogether, these results suggest that girls may pay more attention to social models than boys at an earlier age, adding to interesting and subtle sex differences in the learning of tool use in children^49^.

Finally, our results in both chimpanzees and children are particularly interesting with regards to the notion of a teleological stance^29,31,32^. Our experiment exposed children and chimpanzees to a demonstration from an unfamiliar adult model, who showed the function of each object only twice (each object once in each of two sessions). Our results show that this was enough for around 40% of the children to develop a function-based rule, giving additional support to this theory, but also for one chimpanzee, Ai, to do so as well. This suggests that, contrary to claims that the teleological stance is uniquely human^31,32^, chimpanzees may also learn the function of an object through social observation of a model acting purposefully with this object. In the wild, such ability could assist in acquiring cultural knowledge through social learning, particularly when demonstrators are closely related individuals, whom naïve learners are likely to attend to closely^50,51^.

In conclusion, our study shows that examining conceptual understanding is possible from a comparative perspective in chimpanzees and children if an equally challenging task is presented to both species. We have done so by introducing the MTF task, which modifies standard identity-based MTS to investigate function-based categorisation. Future work should investigate how MTS-inspired paradigms can be extended both conceptually and experimentally so as to precisely tackle the understanding of function and other cognitive evaluations beyond the perceptual level in both humans and non-humans. While we have uncovered the possibility that chimpanzees may be able to categorize their tools based on their functional properties (as was the case in one of our participants), we have also found that they may be more susceptible to perceptual biases compared to children, who were more concerned with the actions of the demonstrator, underscoring the existence of the teleological stance in human development. Nevertheless, our study shows that the roots of this teleological stance must be investigated in our evolutionary past. In particular, future work should strive to isolate a function-based categorization process that can sustain a concept of tool in chimpanzees, and possibly in other species, as a possible building block for the evolutionary origins of our own understanding of tools.

## Supplementary information

Video S1

Supplementary dataset

## Data Availability

All data are available in the Supplementary material.
